# The tug-of-war over MTOR in *Legionella* infections

**DOI:** 10.15698/mic2017.02.559

**Published:** 2017-01-30

**Authors:** Stanimir S. Ivanov

**Affiliations:** 1 Department of Microbiology and Immunology, Louisiana State University Health Sciences Center - Shreveport, Shreveport, LA 71130.

**Keywords:** Legionella, macrophage, lipids, lipogenesis, intracellular pathogens, MTOR, PI3K, SREBP

## Abstract

A ruptured bacteria-containing organelle within the cytosol of an infected eukaryotic cell frequently initiates host defense responses that restrict pathogen replication. Therefore, source for lipids must be found to accommodate the organelle membrane expansion required to support bacterial replication. How host cells are manipulated to provide lipids for the expansion of pathogen-occupied organelles is not well understood. By investigating the interaction between macrophages and the human pulmonary pathogen *Legionella pneumophila* we uncovered that the host metabolic checkpoint kinase Mechanistic target of rapamycin (MTOR) is a central regulator of the pathogen niche expansion program.

Previously, we uncovered that mouse bone marrow-derived macrophages (BMMs) infected with pathogenic *Legionella* suppress MTOR to promote inflammation through a host-driven mechanism. In our recent study (Abshire *et al*., PLoS Pathogens 2016 Dec 12;12(12):e1006088) we identified the Toll-like Receptor (TLR) adaptor Myeloid differentiation primary response gene 88 (Myd88) as a critical factor mediating the host-induced MTOR suppression in *Legionella* infected BMMs. Deletion of Myd88 unmasked an unexpected ability of *L. pneumophila* to activate and sustain MTOR function even in the absence of growth factors or TLR stimulation. BMMs harboring pathogenic *L. pneumophila* elicited robust phosphorylation of the ribosomal S6 protein (a downstream substrate of the MTOR pathway) that was sustained for the entire intracellular replication cycle of the bacterium. In sharp contrast, uninfected neighboring cells remained quiescent (Figure 1A and B). Further investigation revealed that one or more protein(s) encoded by *L. pneumophila* is translocated through the bacterium Type IVb secretion system (T4bSS) directly into the host cytosol to activate MTOR in a phosphatidylinositol-4,5-bisphosphate 3-kinase (PI3K)-dependent manner. The *Legionella* T4bSS manipulates host functions through delivery of ~300 bacterial effector proteins into the host cytosol and is critical for virulence. Thus, it appears that in *L. pneumophila-*infected macrophages the host and the pathogen are locked in a molecular tug-of-war over MTOR function, where suppression favors the host and activation likely favors the pathogen.

**Figure 1:**
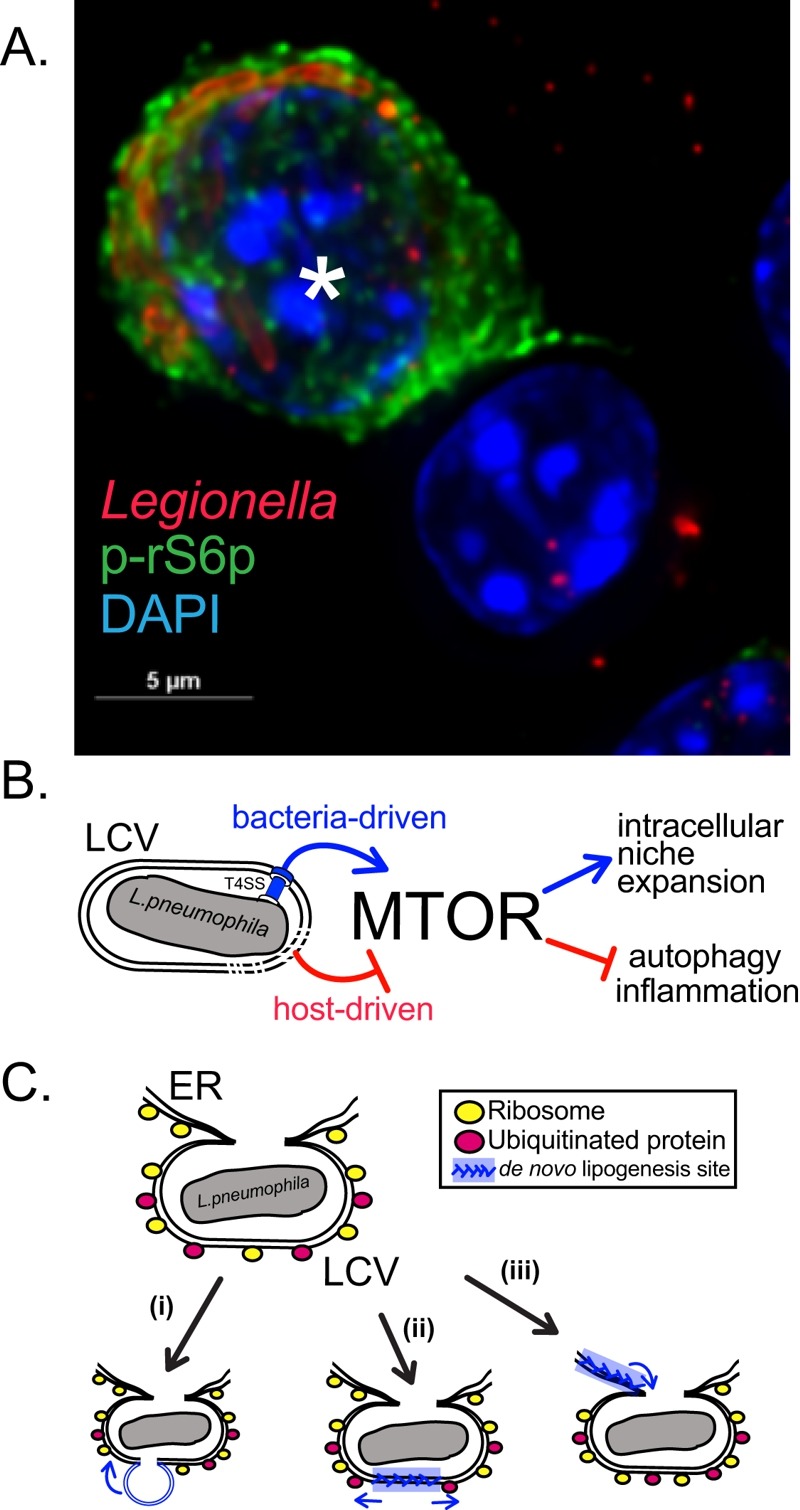
FIGURE 1: MTOR role in *Legionella* macrophage infections. **(A)** A representative micrograph depicting robust MTOR signaling in an infected Myd88^-/-^ BMM (*) but not in an adjacent bystander cell. **(B)** The balance between multiple regulatory mechanisms dictates MTOR signaling in the *Legionella* infected macrophages. **(C)** Putative LCV expansion mechanisms through (i) fusion with membrane-bound organelles, (ii) *de novo* lipogenesis at the LCV or (iii) through ER-derived membrane infusion.

The benefit of sustained MTOR activity for *Legionella* was revealed in part because in MTOR studies cells are generally serum-starved prior to stimulation. Under those conditions, membrane biogenesis requires *de novo* lipogenesis or lipids recycling through autophagy. Blocking MTOR function in *Legionella* infections through various approaches destabilized the *Legionella*-containing vacuole (LCV), led to its premature rupture and the release of bacterial products into the host cytosol, which in turn activated a host cell death response. Culture supplementation with serum but not delipidated serum maintained LCV integrity and blocked the host cell death response implicating a lipids shortage as the likely cause for the loss of LCV integrity under MTOR suppression conditions. Because *Legionella* efficiently blocks autophagy through LC3 delipidation, the role of MTOR-dependent *de novo* lipogenesis was investigated. The transcription factors Serum Response Element Binding Protein 1 and 2 (SREBP1/2) are key lipogenesis regulators downstream of MTOR. *Legionella* infection induced *Srebf1* expression as well as SREBP1/2-regulated genes in infected macrophages. Similarly to MTOR suppression, SREBP1/2 inhibition produced a LCV instability phenotype, which was complemented by dietary lipids. Taken together these data indicates that *Legionella* manipulates the PI3K-MTOR-SREPB1/2 axis to sustain MTOR-dependent lipogenesis to accommodate LCV expansion for optimal intracellular replication.

If dietary lipids and *de novo* lipogenesis are functionally redundant for LCV growth in mammalian macrophages one might wonder why *Legionella* has retained the capacity to activate MTOR? MTOR anabolic regulation is conserved in unicellular amoebae, the natural hosts for *Legionella, *which inhabit aquatic environments and lack constant source of dietary lipids. MTOR inhibition reduced *Legionella* intracellular replication in *Acanthamoeba castellanii* but only moderately. This observation raises the question if *Legionella* could manipulate host lipogenesis independent of MTOR and SREBPs or perhaps LCV expansion can be accommodated from pre-existing membranes (Figure 1C). In principle, fusion with other membrane-bound organelles could facilitate LCVs expansion. Indeed, *Legionella* T4bSS effectors repertoire include proteins that promote recruitment and fusion of the LCV with the early secretory pathway as well as the endoplasmic reticulum. The ER is a major biosynthesis compartment for most cellular lipids and its large membrane reservoir could potentially accommodate some LCV expansion even in the absence of *de novo* lipogenesis. Considering that we identified a number of *Legionella *species that replicate intracellularly but do not activate MTOR it would be important to determine the different mechanisms by which LCV compartments expand.

*Legionella* infections could be an excellent model system to decipher how MTOR controls SREBP1/2 and lipogenesis - a key cellular process that is still poorly understood in primary macrophages. Our work points to a rapamycin-insensitive MTOR-dependent mechanism although additional work is needed to elucidate which MTOR complex is involved and the precise mechanism. Host membranes are key features for successful intracellular survival of vacuolar pathogens and we uncovered that subversion of MTOR by *L. pneumophila* is one mechanism by which the host membrane biogenesis program can be manipulated for niche homeostasis.

